# Validity and reliability of the Patient Health Questionnaire scale (PHQ-9) among university students of Bangladesh

**DOI:** 10.1371/journal.pone.0269634

**Published:** 2022-06-08

**Authors:** Mahir A. Rahman, Tahia Anan Dhira, Abdur Razzaque Sarker, Jeenat Mehareen

**Affiliations:** 1 Bangladesh Institute of Development Studies, Dhaka, Bangladesh; 2 Department of Economics, University of Dhaka, Dhaka, Bangladesh; 3 Department of Economics, East West University, Dhaka, Bangladesh; Universidade Federal do Rio Grande do Sul, BRAZIL

## Abstract

This study investigated the reliability and factorial validity of Patient Health Questionnaire-9 (PHQ-9) in the context of university students in Bangladesh. The research aimed to assess whether the original one-dimensional model or a model containing both somatic and cognitive-emotional factors is appropriate in the case of a sample of university students. A repeated cross-sectional survey design based on convenience sampling was used to collect data from 677 university students from both public and private universities. The factor structure of the PHQ-9 was assessed using confirmatory factor analysis (CFA). Measurement invariances were assessed across gender, type of university, level of education and victim of domestic violence. Its convergent validity was determined by investigating its correlations with Generalized Anxiety Disorder-7 (GAD-7) and Patient Health Questionnaire Anxiety-Depression Scale (PHQ-ADS). Results showed excellent reliability of PHQ-9 as measured by both Cronbach’s α and McDonald’s ω. CFA suggested that a modified one-factor model where the error variances between item-3 (‘sleeping difficulties’) and item-6 (‘feeling as a failure’), item-6 and item-9 (‘suicidal thoughts’), item-4 (‘feeling tired’) and item-9, item-3 and item-9 were allowed to covary is appropriate for the sample. This model provided high values of comparative fit index (CFI), goodness of fit index (GFI), and Tucker Lewis Index (TLI), low value of standardized root mean square residual (SRMR) and a non-significant root mean square error of approximation (RMSEA) as well as a high Factor Determinacy Score Coefficient. Correlation between PHQ-9 and GAD-7 was 0.751 and 0.934 between PHQ-9 and PHQ-ADS. Finally, the model is strictly invariant across gender and university type. Overall, the study provided support for modified unidimensional structure for PHQ-9 and showed high internal consistency along with good convergent validity.

## Introduction

Depression is one of the most common mental disorders, affecting more than 300 million people globally [1]. At its worst, depression can lead to suicide [2, 3]. Furthermore, depressive disorders caused about 50 million Years Lived with Disability (YLD) globally in 2015 [1]. Symptoms of depression is particularly prevalent among university students [4–6]. A study indicated that approximately 82% of university students in Bangladesh were suffering from mild to severe symptoms of depression, a rate which is higher than those reported in the studies conducted on other general population [6–8]. University students go through significant changes in their emotional (e.g., loneliness, personal autonomy) and physical environment (e.g., transition from college to university) [9]. They also deal with the stress of academic competition, plan for their future careers, and become more involved in family matters as adults [10, 11]. Hence, increased vulnerability of university students to symptoms of depression is not unexpected.

Despite high prevalence and potentially severe consequences of including decreased quality of life, multiple somatic complaints, and increased mortality due to suicide, cardiovascular diseases, stroke, obesity morbidity, etc., depression has been largely ignored as a mental health condition [12, 13]. People suffering from depressive disorders are frequently under-diagnosed and/or misdiagnosed, which hinders effective treatment [14–16]. To overcome these obstacles, a reliable, valid, and rapid screening tool for depressive disorders is needed, especially for university students. The nine-item Patient Health Questionnaire scale (PHQ-9) was developed with the purpose of screening for, and assessing the severity of depressive symptoms in both clinical and research contexts [17]. The initial validation research of PHQ-9 demonstrated strong reliability, as well as criteria, concept, and external validity, in a large primary care sample [17]. Subsequently, the psychometric properties of PHQ-9 have been evaluated in different samples of patients, including other primary care samples [18–20], psychiatric patients [21, 22], specific groups of medical patients [23–25] and also in multicultural populations [26–28]. In academic setting, validation of PHQ-9 scale was performed among university students of Korea [29], China [30], Nigeria [31] etc. In the context of Bangladesh, PHQ-9 has been widely used in various studies as a screening tool for depressive symptoms among adolescents, adults and university students [6, 32–35]. However, while Dhira et al. (2021) have explored the psychometric properties of Generalized Anxiety Disorder-7 (GAD-7) for a sample of university students, to the best of our knowledge, no such study has been conducted for the case of PHQ-9 [36].

While studies have found evidence of adequate internal consistency and moderate to strong correlation with other comorbid disorders (anxiety, worry, etc.), findings regarding the factor structure of PHQ-9 have not been consistent. Symptoms of depression can include both psychological symptoms such as: loss of interest and enjoyment, feelings of low self-worth, difficulty in concentrating and making decisions, as well as physiological symptoms such as: changes in appetite and weight unrelated to diet, changes in patterns of sleeping, increased fatigue, increase in purposeless physical activity (e.g., inability to sit still, pacing, handwringing), slowed movements or speech, etc. [37]. Accordingly, some studies showed support for a unidimensional factor structure (containing only a somatic factor) which aligns with the result of the original validation study [20, 29, 38, 39]. In contrast, others indicated a two-dimensional factor structure [40–42], containing both somatic and cognitive-emotional factors. The lack of consensus regarding the factor structure of PHQ-9 in different contexts warrants a comprehensive validation study consisting of Bangladeshi university students.

Against this backdrop, we conducted this study with the objective of investigating the reliability and factorial validity of PHQ-9 on a sample of university students in Bangladesh. The research aimed to evaluate whether the original one-dimensional model or a model containing both somatic and cognitive-emotional factors is appropriate for university students. We also assessed the convergent validity of PHQ-9 with other relevant measures of mental health conditions, namely Generalized Anxiety Disorder-7 (GAD-7) and Patient Health Questionnaire Anxiety-Depression Scale (PHQ-ADS). Finally, after testing for measurement invariance, we examined the mean PHQ-9 scores of the students across different demographic and socioeconomic correlates. We expect that the study will contribute to the growing body of literature pertaining to validation studies assessing symptoms of depressive disorders in university students.

## Material and methods

### Procedure and sampling

A repeated cross-sectional survey was used to collect responses from the university students of Bangladesh. We utilized a snowball sampling strategy in order to capture both public and private university students. Data was collected in two waves: July 18-July 31, 2020 and February 10-February 22, 2021; using the survey Administration software Google Form [43]. To be eligible for the study, the participants had to meet the following criteria: (a) be willing to participate in the study; (b) be enrolled in any public or private university in Bangladesh; (c) have internet access; and (d) be able to read, write, and comprehend the English questionnaire.

Approximately 1.3 million students currently pursue higher education in 47 public and 107 private universities in Bangladesh [44–46]. Considering this population, we calculated the sample size based on the formula:

$$\text{n}=\frac{z^{2}p\left(1-p\right)}{e^{2}}$$

where, n is the sample size, z is the selected critical value of the desired confidence level, p is the estimated proportion of an attribute that is present in the population, and e is the desired level of precision. Using 5% margin of error, 99% confidence level, and 50% response distribution, the sample size was estimated to be 666.

The questionnaire was circulated among two public and three private university students. Students from these universities were most likely to have access to a suitable internet connection and also use English as a mode of learning. Therefore, it was convenient for us to reach them through social media platforms while keeping the questionnaire in its original form. The questionnaire (Google Form link) was initially shared with faculty members of those selected universities, and they were asked to distribute the questionnaire in their respective classrooms either via e-mail or through any course material sharing platform that they were using for communication. We also asked the faculty members to encourage the students to pass on the survey link among their classmates. The final collection of data had a sample of 677 participants studying at different levels of university who responded anonymously to a structured questionnaire which included questions regarding socio-demographic information as well as the items of Patient Health Questionnaire (PHQ-9).

### Description of the instruments

The PHQ-9 is a self- administered version of the Primary Care Evaluation of Mental Disorders (PRIME-MD) diagnostic instrument for common mental health disorders, which is used to determine severity of initial symptoms of depression, and also to monitor symptom changes and treatment effects over time [47]. Participants are asked how often they have encountered symptoms of depression such as: hopelessness, trouble concentrating, etc. during the last two weeks. Response options for each item range from 0 to 3 on a 4-point Likert-scale (0 = not at all, 1 = several days, 2 = more than half the days and 3 = nearly every day). Adding the scores of all nine items provide the PHQ-9 total score differing from 0 to 27. Several validation studies have detected cut-points of ≥ 5, ≥ 10, ≥ 15 and ≥ 20 based on receiver operating characteristics analyses for PHQ-9, standing for mild, moderate, moderately severe and severe depression levels, respectively [48–51].

We have also used the GAD-7 and PHQ-ADS scale to test for convergent validity of PHQ-9. The self- administered seven-item instrument GAD-7 is used as a screening tool to assess the presence and severity of GAD [52–54]. In the assessment, participants are asked how often during the last two weeks they have encountered anxiety symptoms like feeling nervous, trouble relaxing, etc. The range of the scale’s response options and calculation of total score is similar to PHQ-9. S1 Table describes the items and scores of PHQ-9 and GAD-7 questionnaires.

Lastly, the PHQ-ADS is a composite measure that assesses the overall burden of anxiety and depressive symptoms (mental distress) by combining the sum of the PHQ-9 and GAD-7 scores [55]. Thus, the scale can range from 0 to 48, with higher scores indicating higher levels of depression and anxiety symptomatology. Cut points of 10, 20, and 30 on the PHQ-ADS can be considered as thresholds of mild, moderate, and severe distress symptoms, respectively.

### Statistical analysis

Characteristics of the items were examined by exploring item mean score and item-intercorrelations (Table 2, S2 Table). While Cronbach’s α is widely used as a measure of internal consistency and reliability, there are several issues involved. For example, holding the average inter-item correlation constant, α increases if the number of items increase [56]. If the number of items is sufficiently large, α could be large even though the intercorrelation between the items is generally quite small [57, 58] Therefore, α does not directly measure internal consistency or homogeneity of item responses. Furthermore, Cronbach’s α relies on tau-equivalent measurement model, a measurement model that requires a number of assumptions to be met for the estimate to accurately reflect the data’s true reliability [59–61]. Therefore, we use both Cronbach’s α and McDonald’s ω to measure internal consistency and reliability [61] (Table 2).

For applicability purpose, Bartlett Test of Sphericity and Kaiser-Meyer-Olkin (KMO) measure of sampling adequacy was assessed [62]. To analyze construct validity of PHQ-9, confirmatory factor analysis (CFA) was performed with structural equation model (SEM) [63, 64]. Using CFA, we tested four alternative models for the structure of the PHQ-9 to understand the dimensionality of the scale [29]. Model 1 is the original one-factor model suggested by Kroenke et al. [17]. Model 2 is a two-factor model suggested by Krause et al. [65], where the items ‘sleeping difficulties’, ‘fatigue’ and ‘appetite change’ get loaded on a somatic factor. Model 3 is another two-factor model derived by Richardson and Richard [66], where the somatic factor also includes ‘concentration difficulties’ and ‘retardation/slowed down speech and movement’ along with the three items suggested in Model 2. Model 4 is the modified one factor model suggested by inspection of the modification indices in our study.

Next, these models were compared using several model fit indices and their criteria, including (i) the chi-square (*χ*^2^) and its degrees of freedom (df), (ii) root mean square error of approximation (RMSEA) and its 90% confidence interval, (iii) comparative fit index (CFI), (iv) goodness of fit index (GFI), (v) Tucker Lewis Index (TLI) and (vi) standardized root mean square residual (SRMR) (Table 3). RMSEA values of less than or equal to 0.05 represents close fit, while values between 0.05 to 0.08 are considered acceptable fit [67, 68]. GFI values greater than 0.9 indicate good fit [69]. CFI [70] and TLI [71] are incremental fit indices and values of greater than or equal to 0.95 of these indices indicate very good fit [72] and values of 0.90 or above are considered acceptable fit [73]. SRMR values up to 0.05 indicate close-fit, while values between 0.05 to 0.10 suggest acceptable fit [73]. We also tested factor score determinacy coefficient to evaluate the goodness of fit of the models. According to Gorsuch (1983) this coefficient should be > = 0.90 if the factor score is to be used as a substitute for the factor itself [74].

In order to utilize the PHQ-9 for meaningful comparisons in depressive symptoms across different socioeconomic groups, we tested whether measurement invariance holds across these groups [75]. Thus, we carried out multiple-group confirmatory factor analysis based on the unidimensional modified model between two gender groups (male, female), two types of university students (public, private), five different years of education levels in the university and whether the respondent was a victim of domestic violence to investigate if PHQ-9 assesses the same construct across these groups and that observed differences in PHQ-9 scores among these groups reflect true group differences in depressive symptoms. The first one of the four increasingly constrained CFA models had all parameters free (configural invariance). The second one took equal loadings (weak invariance), while the third model required equal loadings and intercepts (strong invariance). Lastly, the fourth one is the most constrained model with equal loadings, residuals, and intercepts (strict invariance). The essential criterion for comparing models with additional constraints were the change in CFA and RMSEA. ΔCFI <0.01 and ΔRMSEA < 0.015 support for measurement invariance [76].

To assess convergent validity of the PHQ-9, the association between PHQ-9 and the Generalized Anxiety Disorder (GAD-7) and Patient Health Questionnaire Anxiety and Depression Scale (PHQ-ADS) were examined using Pearson’s correlation (r) and its significance. Mean scores of PHQ-9 index across sample characteristics for which measurement invariance holds were also studied using t test and analysis of variance (ANOVA) (Table 4).

Data cleaning, validation, and all statistical analyses were performed using Stata/IC 16.1 (StataCorp, College Station, TX, USA) and R studio, with the packages ‘lavaan’, ‘semTools’, and ‘psych’.

### Ethical considerations

Ethical permission for data collection was taken from respective faculty and department heads of the universities where the questionnaire was distributed. All participants gave their informed consent to anonymously (unidentified to the authors) participate in the study. In the consent form, participants were provided with information concerning the purpose, procedure and nature of the study, the option to take part as well as the right to revoke their data at any point of the study. The research is approved by the Department of Economics, East West University and procedures of this study complied with the provisions of the Declaration of Helsinki (1989) regarding research on human participants.

## Results

Table 1 shows the distributions of the key socio-demographic variables. Males made up 51.40% of the 677 participants, while females made up 48.60%., with public university students accounting for over a third of the sample (65.19%). Distribution of PHQ-9 items are represented in S3 Table.

**Table 1:** Socio-demographic characteristics.

Variables	Categories	N	% in the sample
**Age**	18–22 years	550	81.24
	23–27 years	127	18.76
**Gender**	Male	348	51.40
	Female	329	48.60
**Education Level**	First year	309	45.64
	Second year	98	14.48
	Third year	131	19.35
	Fourth year	95	14.03
	Masters	44	6.50
**University Type**	Public University	440	65.19
	Private University	235	34.81
**Marital Status**	Married	8	1.19
	Single	661	97.93
	Others	6	0.89
**Student employment**	Yes	102	15.07
	No	575	84.93
**Family monthly income**	<25,000 BDT	154	22.75
	25,000–54,999 BDT	250	36.93
	55,000–99,999 BDT	174	25.70
	> = 1,00,000 BDT	99	14.62
**Principal Income Source**	Government Service Holder	189	27.92
	Agricultural wage labor	35	5.17
	Organized Trade/Business	173	25.55
	Pension/ Rent	87	12.85
	Private Service Holder	181	26.74
	Others	12	1.77
**Joint Family**	No	536	79.17
	Yes	141	20.83
**Family Size**	< = 4 members	340	50.22
	>4 members	337	49.78
**Majority of time spent**	Alone	191	28.21
	With family	409	60.41
	With friends	66	9.75
	With pets	11	1.62
**Domestic violence**	Yes	96	14.18
	No	581	85.82
**Victim of domestic violence**	Yes	65	67.71
	No	31	32.29

### Item characteristics

Item characteristics are summarized in Table 2. The highest reported score is on item-3 ‘Trouble falling asleep or sleeping too much’ (1.58 ± 1.13) and the lowest reported score is on item-9 ‘Thoughts of dying, or hurting self’ (0.64 ± 0.99). Correlation between the items were significant and moderate enough (ranging from 0.10–0.51) to justify conducting factor analysis [77, 78] (S2 Table).

**Table 2:** Characteristics of items and total PHQ-9 scale.

PHQ-9 Items	Mean (95% CI)	SD	Factor Loadings	Cronbach’s α	McDonald’s ω
1. Little interest or pleasure in doing things?	1.27 (1.19–1.34)	0.96	0.276	0.824	0.86
2. Feeling down, depressed, or hopeless?	1.44 (1.36–1.53)	1.08	0.749		
3. Trouble falling or staying asleep, or sleeping too much?	1.58 (1.50–1.67)	1.13	0.653		
4. Feeling tired or having little energy?	1.39 (1.31–1.47)	1.06	0.743		
5. Poor appetite or overeating?	1.03 (0.95–1.11)	1.08	0.673		
6. Feeling bad about yourself—or that you are a failure or have let yourself or your family down?	1.31(1.22–1.40)	1.16	0.730		
7. Trouble concentrating on things, such as reading the newspaper or watching television?	1.28 (1.19–1.37)	1.16	0.663		
8. Moving or speaking so slowly that other people could have noticed?	0.78 (0.71–0.85)	0.99	0.660		
9. Thoughts that you would be better off dead, or of hurting yourself in some way?	0.64 (0.57–0.72)	0.99	0.588		
PHQ-9 Total Score	10.74 (10.27–11.20)	6.20	___		

CI = Confidence Interval; SD = Standard Deviation.

### Reliability

The value of the reliability coefficient Cronbach’s α and McDonalds’ ω for the overall PHQ-9 scale are 0.824 and 0.86 respectively, which is greater than the recommended value of 0.80, indicating excellent reliability [79]. (Table 2).

### Construct validity

Construct validity of the scale was tested with confirmatory factor analysis. Applicability of factor analysis was tested using KMO and Bartlett Test of Sphericity. The KMO coefficient is 0.879 surpasses the recommended value of 0.6, while Bartlett Test of Sphericity is found statistically significant (χ^2^ = 1689.151, df = 36, p<0.001), indicating the suitability of performing factor analysis on this sample [79]. All the items of PHQ-9 have statistically significant loadings (p<0.001). Therefore, all seven items of the measure are important to interpret (Table 2).

We performed CFA on four alternative models. CFA does not satisfy adequate fit criteria for the original one-factor model (Model 1) (Table 3). While the value of SRMR is less than 0.05, the chi-square value is significant at p<0.001 suggesting poor fit. Besides, chi-square provides inflated value when sample size is large and does not work well where sample size is small, and the underlying distribution may be non-normal [80]. Moreover, CFI, GFI and TLI values are less than 0.950, again indicating poor fit. RMSEA value is comparatively higher and statistically significant (p<0.001) suggesting unacceptable fit (Table 3).

**Table 3:** Goodness of fit indices for the PHQ-9 item factor models (N = 677).

Model	k	*χ* ^2^	df	CFI	GFI	TLI	RMSEA (90% CI)	SRMR
Model 1	9	153.823***	27	0.924	0.910	0.898	0.083***(0.071–0.096)	0.046
Model 2	9	113.820***	26	0.947	0.933	0.927	0.071**(0.058–0.084)	0.040
Model 3	9	108.974***	26	0.950	0.936	0.931	0.069** (0.056–0.082)	0.040
Model 4	9	74.740***	23	0.969	0.956	0.951	0.058 (0.043–0.073)	0.033

***p<0.001;

**p<0.05;

*p< 0.1

k = number of items; df = degrees of freedom; CFI = comparative fit index; GFI = goodness of fit index; TLI = Tucker-Lewis index; RMSEA = root mean squared error of approximation; SRMR = standardized root mean residual

Model 1: Originally validated one- factor model

Model 2: Two- factor model of Krause et al. (2008) with item 3, 4 and 5 loaded on one somatic factor and the other six items loaded on an affective factor

Model 3: Two- factor model of Richardson and Richard with item 3,4,5,7,8 loaded on the somatic factor and the others on affective factor

Model 4: Modified one-factor model.

Goodness of fit indices for both of the two-factor models also do not meet the required cut off values. Consequently, a modification was conducted to improve the values of goodness of fit indices for the original one-factor model and the error variances between item-3 (‘sleeping difficulties’) and item-6 (‘feeling as a failure’), item-6 and item-9 (‘suicidal thoughts’), item-4 (‘feeling tired’) and item-9, item-3 and item-9 were combined to construct Model 4. Modified one-factor model provides non-significant and the lowest RMSEA, the highest and acceptable values of CFI, GFI and TLI which are all greater than the required cut off score 0.950 [72] and the lowest value of SRMR (0.033) [81]. Furthermore, Factor Determinacy Coefficient suggested that the modified unidimensional model is the most well-defined for this sample (S4 Table).

### Convergent validity

Convergent validity of the PHQ-9 was determined by its Pearson’s correlations with other measures used in the study. Scores of the PHQ-9 scale were highly and positively correlated with the scores of GAD-7 and PHQ-ADS. Correlation between PHQ-9 and GAD-7 is 0.751 and between PHQ-9 and PHQ-ADS is 0.934. Both the correlations are statistically significant (p<0.001) (S2 Table).

**Fig 1:**
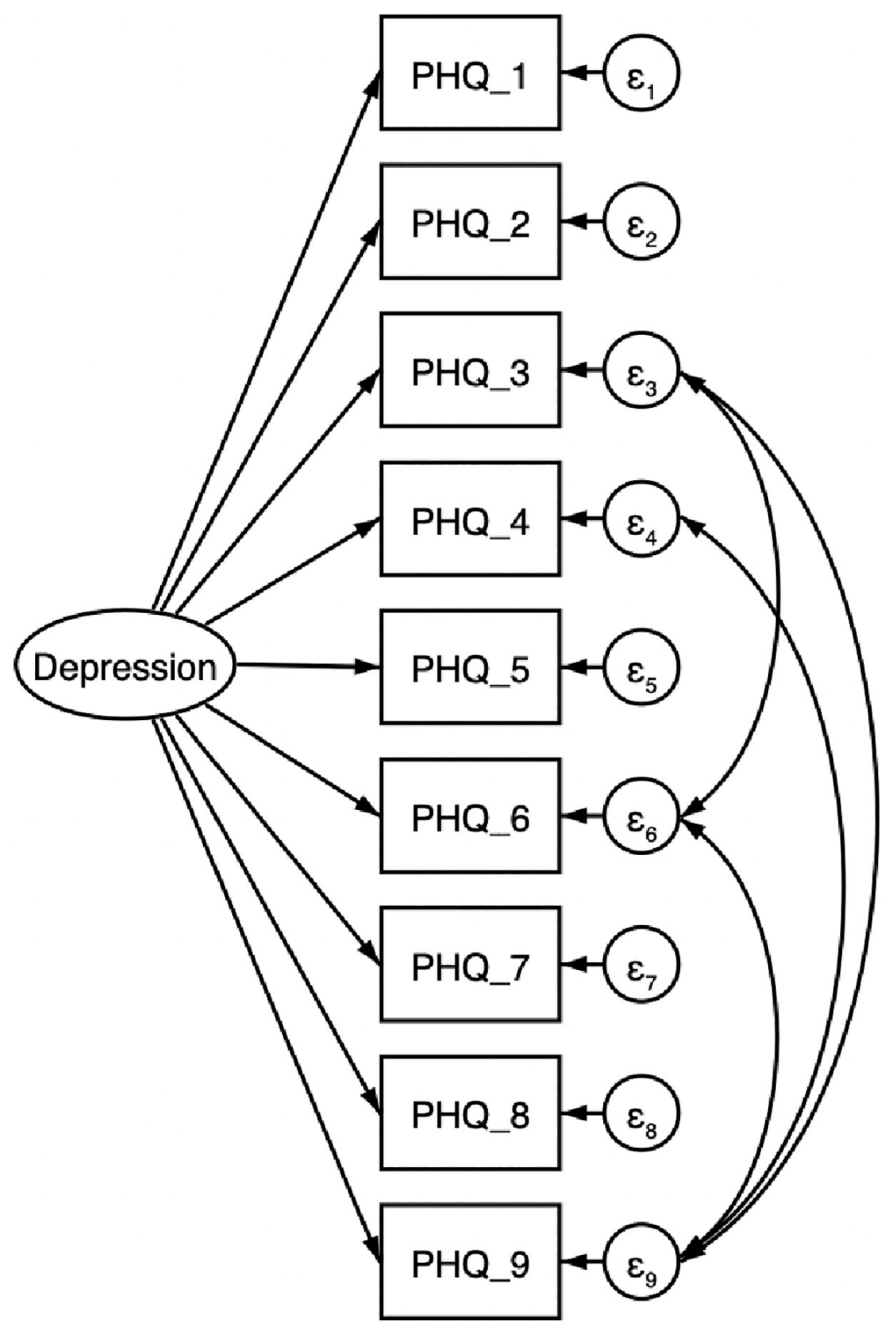
Confirmatory factor analysis path diagram for modified one-factor model of PHQ-9 factors. (All factor loadings and error covariances were significant at p<0.001).

Thus, we conclude that the modified one factor model is the best fit to the data for our sample. All factor loadings and error covariances are statistically significant (p<0.001), suggesting that the indicator variables are significantly related to their respective factor. Confirmatory factor analysis path diagram is represented in Fig 1 and the fit indices are shown in Table 3.

### Measurement invariance

Configural, weak and strong invariance models for both gender and university type had statistically insignificant Chi-square, suggesting strong invariance. The strict invariance model for gender and university type were significant at 5% level, according to Chi-square difference. However, as it in highly perceptive to the sample size and minor mis-specification can result in substantial Chi-square difference [82], we emphasize on differences of CFI and RMSEA. All models with progressively stronger constraints exhibited ΔCFI <0.01 and ΔRMSEA < 0.015, for gender and type of university, suggesting strict invariance (S5 Table). However, victim of domestic violence in the family and level of education are weakly invariant as the Chi-square differences were significant and also the ΔCFI was not less than 0.01 for strong invariant model. These results lend sufficient support for a comparison of mean PHQ-9 scores across these socioeconomic groups of our sample.

### PHQ-9 scores across socio-demographic characteristics

Table 4 shows the responsiveness of PHQ-9 index over the values of the variables those are important for our study and have been used to test measurement invariance. From the table, we can see students who are female, studying in public university, and faced domestic violence in the family have significantly stronger symptoms of depression. We also found significantly higher PHQ-9 score for students who are enrolled in third and fourth year of undergraduate studies.

**Table 4:** Association of PHQ-9 score with socio-demographic characteristics (N = 677).

Variables	Categories	PHQ-9 score, Mean (SD)	t/F	P-value
**Gender**	Male	9.61 (6.01)	4.922	**0.000**
	Female	11.92 (6.17)		
**Type of University**	Public University	11.58 (6.25)	-5.068	**0.000**
	Private University	9.09 (5.74)		
**Level of Education**	First year	9.74 (5.95)	5.46	**0.000**
	Second year	10.48 (6.12)		
	Third year	12.38 (6.47)		
	Fourth year	11.99 (6.41)		
	Masters	10.68 (5.45)		
**Domestic violence in family**	Yes	12.82 (6.71)	-3.594	**0.000**
	No	10.39 (6.04)		

*Group differences were performed using t test and analysis of variance. Significant group differences are printed in bold (P <0.05)

SD = Standard Deviation.

## Discussion

PHQ-9 has been used to detect symptoms of depressive disorders across diverse populations, beyond its original application in primary-care settings. However, a paucity of studies conducted on vulnerable groups such as university students necessitates a contribution to the existing gap in the literature. In this context, our study examined the psychometric properties of the PHQ-9 on a sample of university students in Bangladesh, using CFA.

Internal consistency of the scale was excellent in our sample, reflected by the overall Cronbach’s α of 0.824. To conclude regardless of tau-equivalence assumption, we have also computed McDonald’s ω [61], which is 0.86, ensuring reliability of the scale in our sample. The original validation study conducted on 3000 primary care patients and 3000 ob-gyn patients found excellent internal reliability of PHQ-9, with a Cronbach’s α of 0.89 and 0.86 respectively [17]. Later, clinical studies in Chile [83], England [41], Germany [39], United States [84], Iran [85], and China [64] in addition to non-clinical studies conducted in Ghana [86], Hong Kong [87], Philippines [88] revealed the evidence of good internal consistency of PHQ-9 scale across different populations through excellent Cronbach’s α coefficient. Specifically, studies conducted on university students from Korea (Cronbach’s α = 0.83) [29], Nigeria (Cronbach’s α = 0.85) [31] and China (Cronbach’s α = 0.80) [30] found excellent internal consistency of PHQ-9.

We tested the convergent validity of PHQ-9 with two other scales, GAD-7 and PHQ-ADS. Correlation coefficient between PHQ-9 and GAD-7 and, between PHQ-9 and PHQ-ADS were significant and greater than 0.75, suggesting satisfaction of convergent validity (S2 Table). Previous studies have observed the comorbidity of PHQ-9 with anxiety disorders [89–91]. The study on Korean university students has also found good convergent validity of PHQ-9 with GAD-7, with a correlation coefficient of 0.68 [92]. Other studies have also found strong evidence of convergent validity of PHQ-9 with similar psychometric instruments in different settings [29, 31, 47, 93–98]. Together, these findings suggest the reliability and validity of applying the PHQ-9 scale as a measure of symptoms of depression in the context of university students in different countries.

The unidimensional model showed a marginal fit to our context. The original model was therefore revised using the examination of modification indices. Dependency of the error variances between item-3 (‘sleeping difficulties’) and 6 (‘feeling as a failure’), 6 and 9 (‘suicidal thoughts’), 4 (‘feeling tired’) and 9 and, 3 and 9 upgraded the fitness of the model. Our modified one-factor model was partially similar to that of Maroufizadeh et al. (2019) for patients with infertility [85] where they found covariance between Item 1 and Item 2 as well as between Item 7 and Item 8, and Item 6 and Item 9. Furthermore, Beards et al. (2016) found evidence for covariance between item 7 (concentration difficulty) and item 8 (motor slowing/restlessness) in case of a two-factor model [22]. On the other hand, Kim & Lee (2019) found support for a one-factor model in the case of Korean university students [29]. A number of studies have also suggested two-factor models with a cognitive and a physical latent factor [40, 65, 66, 99, 100]. However, the modified one-factor model provided the best values of all the goodness of fit indices as well as in terms of factor score determinacy index (S4 Table). This may be due to our non-clinical sample consisting of university students. In contrast, the studies concluding a somatic and a physical factor for PHQ-9 are mainly conducted with clinical populations such as patients with infertility, spinal cord injury etc. [85, 63].

Aside from the issues discussed above, the mean PHQ-9 scores across different sample characteristics were compared with similar analyses from existing literature on university students. As a prerequisite, we tested measurement invariance of the scale across gender, type of university, victim of domestic violence and level of education. Strict invariances were observed for gender, that is consistent with the outcomes of some previous research [20, 38, 101]. In contrast, other studies find no or weak measurement invariance across gender [102]. In the context of Bangladesh, examining measurement invariance across public and private university students is also important [5]. Additionally, level of education and victim of domestic violence were found weakly invariant in our study. Previous research primarily based on adult population found level of education to be strictly invariant [20, 75]. Similar results were also obtained for domestic violence [103]. However, the nature of non-clinical student sample could be the reason behind the absence of strong invariance across education level and domestic violence in our study.

As measurement invariance was established, it is meaningful to discuss differences in PHQ-9 scores across the specific sociodemographic groups of university students. In terms of gender, our results show that higher PHQ-9 scores were associated with female students, in line with the findings of other studies [104, 105]. In case of level of education, we observe that students enrolled in higher level of their undergraduate study have significantly higher PHQ-9 scores. Advanced undergraduate students often need to deal with factors such as failure in love affairs, lack of self-confidence, job and financial insecurity and familial problems. All these factors might contribute to low self-esteem which is associated with increasing depression [106]. The results obtained from our sample also show that the PHQ-9 scores are significantly higher for students from public universities. As public university students in Bangladesh mostly come from a poorer socio-economic background compared to private university students, they have an additional pressure of finding jobs just after or even during their study. As a result, fear of delayed completion of degree and uncertainty of jobs are likely to be contributing factors to the high score [5]. Our results also indicate that students who witnessed domestic violence in the family suffer more from depression compared to those who did not [107, 108].

The study has several limitations which should be considered before interpreting the results. First, the data collected through web-based platform captured a relatively homogeneous sample of students characterized by high literacy and easy internet access, potentially resulting in selection bias. Hence, the findings of this study cannot be generalized across other populations such as older adults, adolescents, patients, etc. The nature of non-clinical student sample could be the reason behind the absence of strong invariance across education level and domestic violence. Second, self-reported mental health metrics such as PHQ-9 might be unduly affected by reporting bias [109, 110]. Lastly, sensitivity and specificity of PHQ-9 for university students should be explored in future research.

## Conclusion

This is the first study to evaluate the psychometric properties of PHQ-9 in university students of Bangladesh and hence contributes to minimize a major gap in the literature. The study adds to the growing evidence of PHQ-9 as a concise, simply administered self-reported questionnaire. The results also provide support for a modified unidimensional structure of PHQ-9 and show high internal consistency as well as good convergent validity for the sample. Such successful validation of PHQ-9 scale in the context of university students of Bangladesh will allow early diagnosis and treatment, thus helping the policy makers and public health authorities to take necessary and timely interventions to combat the prevalence of such disorders.

## Supporting information

S1 Table PHQ-9 and GAD-7 items and scores. (DOCX)

S2 Table Pearson’s correlation coefficients (r) between PHQ-9 items and with other questionnaires, (*n* = 677). (DOCX)

S3 Table Distribution of PHQ-9 items. (DOCX)

S4 Table Factor determinacy coefficients. (DOCX)

S5 Table Multigroup—CFA: Fit measures of the invariance test. (DOCX)

S1 Dataset PHQ-9_dataset. (DTA)
