# Supplementary material for: Validity and reliability of the Patient Health Questionnaire scale (PHQ-9) among university students of Bangladesh
Source: PLoS One. 2022 Jun 8;17(6):e0269634. doi: 10.1371/journal.pone.0269634 (PMC9176811; doi:10.1371/journal.pone.0269634)
Supplement: S1 Table — (DOCX) [file pone.0269634.s001.docx]

| **Patient Health Questionnaire- 9 items (PHQ-9)** | | | | |
| --- | --- | --- | --- | --- |
| **Over the last 2 weeks, how often have you been bothered by any of the following problems?** | **Not at all (0 days)** | **Several days**  **(1-5 days)** | **More than half the days**  **(6-10 days)** | **Nearly every day**  **(11-14 days)** |
| 1. Little interest or pleasure in doing things? | 0 | 1 | 2 | 3 |
| 2. Feeling down, depressed, or hopeless? | 0 | 1 | 2 | 3 |
| 3. Trouble falling or staying asleep, or sleeping too much? | 0 | 1 | 2 | 3 |
| 4. Feeling tired or having little energy? | 0 | 1 | 2 | 3 |
| 5. Poor appetite or overeating? | 0 | 1 | 2 | 3 |
| 6. Feeling bad about yourself - or that you are a failure or have let yourself or your family down? | 0 | 1 | 2 | 3 |
| 7. Trouble concentrating on things, such as reading the newspaper or watching television? | 0 | 1 | 2 | 3 |
| 8. Moving or speaking so slowly that other people could have noticed? | 0 | 1 | 2 | 3 |
| 9. Thoughts that you would be better off dead, or of hurting yourself in some way? | 0 | 1 | 2 | 3 |
| **Generalized Anxiety Disorder – 7 items (GAD-7)** | | | | |
| **Over the last 2 weeks, how often have you been bothered by any of the following problems?** | **Not at all (0 days)** | **Several days**  **(1-5 days)** | **More than half the days**  **(6-10 days)** | **Nearly every day**  **(11-14 days)** |
| 1. Feeling nervous, anxious or on edge? | 0 | 1 | 2 | 3 |
| 2. Not being able to stop or control worrying? | 0 | 1 | 2 | 3 |
| 3. Worrying too much about different things? | 0 | 1 | 2 | 3 |
| 4. Trouble relaxing? | 0 | 1 | 2 | 3 |
| 5. Being so restless that it is hard to sit still? | 0 | 1 | 2 | 3 |
| 6. Becoming easily annoyed or irritable? | 0 | 1 | 2 | 3 |
| 7. Feeling afraid as if something awful might happen? | 0 | 1 | 2 | 3 |

**S1 Table: PHQ-9 and GAD-7 items and scores**
