# Supplementary material for: Validity and reliability of the Patient Health Questionnaire scale (PHQ-9) among university students of Bangladesh
Source: PLoS One. 2022 Jun 8;17(6):e0269634. doi: 10.1371/journal.pone.0269634 (PMC9176811; doi:10.1371/journal.pone.0269634)
Supplement: S2 Table — (DOCX) [file pone.0269634.s002.docx]

**S2 Table: Pearson correlation coefficients (r) between PHQ-9 items and with other questionnaires, (N= 677)**

|  | **PHQ-9** |  |  |  |  |  |  |  |  |
| --- | --- | --- | --- | --- | --- | --- | --- | --- | --- |
| **GAD-7** | **0.751***** |  |  |  |  |  |  |  |  |
| **PHQ-ADS** | **0.937***** |  |  |  |  |  |  |  |  |
|  | **PHQ-1** | **PHQ-2** | **PHQ-3** | **PHQ-4** | **PHQ-5** | **PHQ-6** | **PHQ-7** | **PHQ-8** | **PHQ-9** |
| **PHQ-1** | 1.000 |  |  |  |  |  |  |  |  |
| **PHQ-2** | 0.178******* | 1.000 |  |  |  |  |  |  |  |
| **PHQ-3** | 0.135*** | 0.442*** | 1.000 |  |  |  |  |  |  |
| **PHQ-4** | 0.121** | 0.511*** | 0.500*** | 1.000 |  |  |  |  |  |
| **PHQ-5** | 0.128*** | 0.371*** | 0.443*** | 0.461*** | 1.000 |  |  |  |  |
| **PHQ-6** | 0.107* | 0.513*** | 0.328*** | 0.469*** | 0.392*** | 1.000 |  |  |  |
| **PHQ-7** | 0.207*** | 0.399*** | 0.372*** | 0.412*** | 0.372*** | 0.399*** | 1.000 |  |  |
| **PHQ-8** | 0.104* | 0.380*** | 0.307*** | 0.396*** | 0.394*** | 0.412*** | 0.413*** | 1.000 |  |
| **PHQ-9** | 0.166*** | 0.437*** | 0.196*** | 0.284*** | 0.275*** | 0.481*** | 0.255*** | 0.365*** | 1.000 |

PHQ-9= Patient Health Questionnaire-9, GAD-7= Generalized Anxiety Disorder-7, PHQ-ADS= Patient Health Questionnaire Anxiety and Depression Scale
***p<0.001; **p<0.05; *p<0.1
