# Supplementary material for: Validity and reliability of the Patient Health Questionnaire scale (PHQ-9) among university students of Bangladesh
Source: PLoS One. 2022 Jun 8;17(6):e0269634. doi: 10.1371/journal.pone.0269634 (PMC9176811; doi:10.1371/journal.pone.0269634)
Supplement: S3 Table — (DOCX) [file pone.0269634.s003.docx]

**S3 Table: Distribution of PHQ-9 items**

| **PHQ-9 items** | **Not at all** | **Several days** | **More than half the days** | **Nearly everyday** |
| --- | --- | --- | --- | --- |
| 1. Little interest or pleasure in doing things? | 21.71% | 44.17% | 19.94% | 14.18% |
| 2. Feeling down, depressed, or hopeless? | 22.30% | 34.27% | 20.09% | 23.34% |
| 3. Trouble falling or staying asleep, or sleeping too much? | 22.75% | 24.67% | 23.93% | 28.66% |
| 4. Feeling tired or having little energy? | 22.75% | 36.93% | 18.46% | 21.86% |
| 5. Poor appetite or overeating? | 41.95% | 27.62% | 15.81% | 14.62% |
| 6. Feeling bad about yourself - or that you are a failure or have let yourself or your family down? | 32.79% | 27.03% | 16.40% | 23.78% |
| 7. Trouble concentrating on things, such as reading the newspaper or watching television? | 34.12% | 26.59% | 16.54% | 22.75% |
| 8. Moving or speaking so slowly that other people could have noticed? | 52.88% | 25.55% | 12.26% | 9.31% |
| 9. Thoughts that you would be better off dead, or of hurting yourself in some way? | 63.37% | 18.46% | 8.57% | 9.60% |
