# Supplementary material for: Validity and reliability of the Patient Health Questionnaire scale (PHQ-9) among university students of Bangladesh
Source: PLoS One. 2022 Jun 8;17(6):e0269634. doi: 10.1371/journal.pone.0269634 (PMC9176811; doi:10.1371/journal.pone.0269634)
Supplement: S4 Table — (DOCX) [file pone.0269634.s004.docx]

**S4 Table: Factor Determinacy Coefficients**

| **Models** | **Latent Factors** | **Factor determinacy coefficient** |
| --- | --- | --- |
| Model 1: original one factor model | Depression | 0.921 |
| Model2: Two- factor model with item 3, 4 and 5 loaded on one somatic factor and the other six items loaded on an affective factor | Somatic Depression | 0.897 |
|  | Affective Depression | 0.911 |
| Model 3: Two- factor model with item 3,4,5,7,8 loaded on the somatic factor and the others on affective factor | Somatic Depression | 0.909 |
|  | Affective Depression | 0.900 |
| Model 4: Modified one-factor model | Depression | 0.932 |
