# Supplementary material for: Validity and reliability of the Patient Health Questionnaire scale (PHQ-9) among university students of Bangladesh
Source: PLoS One. 2022 Jun 8;17(6):e0269634. doi: 10.1371/journal.pone.0269634 (PMC9176811; doi:10.1371/journal.pone.0269634)
Supplement: S5 Table — (DOCX) [file pone.0269634.s005.docx]

**S5 Table: Multigroup - CFA: Fit measures of the invariance test**

| **Group** |  | ${}^{\boldsymbol{2}}$ | **df** | **CFI** | **TLI** | **RMSEA** | **SRMR** | **∆**${}^{\boldsymbol{2}}$ | **∆CFI** | **∆RMSEA** |
| --- | --- | --- | --- | --- | --- | --- | --- | --- | --- | --- |
| Gender | Configural | 102.18*** | 46 | 0.965 | 0.946 | 0.060 | 0.036 | - | - | - |
|  | Weak | 108.98*** | 54 | 0.966 | 0.955 | 0.055 | 0.044 | 6.80 | 0.001 | 0.005 |
|  | Strong | 117.53*** | 62 | 0.966 | 0.960 | 0.051 | 0.046 | 8.54 | 0.000 | 0.004 |
|  | Strict | 138.19*** | 71 | 0.958 | 0.958 | 0.053 | 0.052 | 20.67* | 0.008 | 0.002 |
| Type of University | Configural | 99.76*** | 46 | 0.967 | 0.948 | 0.059 | 0.046 | - | - | - |
|  | Weak | 105.68*** | 54 | 0.968 | 0.957 | 0.053 | 0.051 | 5.92 | 0.001 | 0.006 |
|  | Strong | 119.78*** | 62 | 0.964 | 0.958 | 0.052 | 0.054 | 14.08 | 0.004 | 0.001 |
|  | Strict | 137.63** | 71 | 0.959 | 0.958 | 0.053 | 0.059 | 17.88* | 0.005 | 0.001 |
| Education Level | Configural | 200.04*** | 115 | 0.949 | 0.920 | 0.074* | 0.047 | - | - | - |
|  | Weak | 228.78*** | 147 | 0.951 | 0.939 | 0.064 | 0.061 | 28.74 | 0.002 | 0.010 |
|  | Strong | 227.4*** | 179 | 0.941 | 0.940 | 0.064 | 0.069 | 48.62* | 0.010 | 0.000 |
|  | Strict | 326.46*** | 215 | 0.933 | 0.944 | 0.062 | 0.080 | 49.07 | 0.008 | 0.002 |
| Domestic Violence  in family | Configural | 90.54*** | 46 | 0.973 | 0.957 | 0.053 | 0.031 | - | - | - |
|  | Weak | 103.61*** | 54 | 0.970 | 0.959 | 0.052 | 0.039 | 13.15 | 0.003 | 0.001 |
|  | Strong | 139.01*** | 62 | 0.953 | 0.945 | 0.061 | 0.045 | 35.40*** | 0.017 | 0.009 |
|  | Strict | 174.68*** | 71 | 0.937 | 0.936 | 0.066 | 0.055 | 35.67*** | 0.016 | 0.005 |

Note: *** = p <0.001, ** = p<0.01and * = p<0.05
